# Green Synthesis and Antibacterial Effect of Silver Nanoparticles Using *Vitex Negundo* L.

**DOI:** 10.3390/molecules16086667

**Published:** 2011-08-08

**Authors:** Mohsen Zargar, Azizah Abdul Hamid, Fatima Abu Bakar, Mariana Nor Shamsudin, Kamyar Shameli, Fatemeh Jahanshiri, Farah Farahani

**Affiliations:** 1 Department of Food Science, Faculty of Food Science and Technology, Universiti Putra Malaysia, Selangor 43400, Malaysia; E-Mails: zmohsen2002@gmail.com (M.Z.); fatim@putra.upm.edu.my (F.A.B.); 2 Department of Biology, Qom Branch, Islamic Azad University, Qom, Iran; E-Mail: farahfarahani2000@yahoo.com; 3 Faculty of Biotechnology and Biomolecular Science, Universiti Putra Malaysia, Selangor 43400, Malaysia; E-Mail: f.jahanshiri@gmail.com; 4 Department of microbiology and parasitology, Faculty of Medicine, Universiti Putra Malaysia, Selangor 43400, Malaysia; E-Mail: mariana@medic.upm.edu.my; 5 Department of Chemistry, Faculty of Science, Universiti Putra Malaysia, Serdang, Selangor 43400, Malaysia; E-Mail: kamyarshameli@gmail.com; 6 Agro-Biotecnologi Institute Malaysia, Ministry of Science, Technology and Innovation of Malaysia, Selangor 43400, Malaysia

**Keywords:** green synthesis, silver nanoparticles, *Vitex negundo*, antibacterial activity, transmission electron microscopy

## Abstract

Different biological methods are gaining recognition for the production of silver nanoparticles (Ag-NPs) due to their multiple applications. One of the most important applications of Ag-NPs is their use as an anti-bacterial agent. The use of plants in the synthesis of nanoparticles emerges as a cost effective and eco-friendly approach. In this study the biosynthesis of silver nanoparticles using *Vitex negundo* L. extract and its antimicrobial properties has been reported. The resulting silver particles are characterized using transmission electron microscopy (TEM), X-ray diffraction (XRD) and UV–Visible (UV-Vis) spectroscopic techniques. The TEM study showed the formation of silver nanoparticles in the 10–30 nm range and average 18.2 nm in size. The XRD study showed that the particles are crystalline in nature, with a face centered cubic (fcc) structure. The silver nanoparticles showed the antimicrobial activity against Gram positive and Gram negative bacteria. *Vitex negundo* L. was found to display strong potential for the synthesis of silver nanoparticles as antimicrobial agents by rapid reduction of silver ions (Ag^+^ to Ag^0^).

## 1. Introduction 

Nanoparticles are often referred to as particles with a maximum size of 100 nm. Nanoparticles exhibit unique properties, which are quite different than those of larger particles. New properties of nanoparticles related to variation in specific characteristics like size, shape and distribution have been demonstrated [1]. Among the noble metals (e.g., Ag, Pt, Au and Pd), silver (Ag) is the metal of choice for potential applications in the field of biological systems, living organisms and medicine [2]. Due to their exclusive properties, silver nanoparticles (Ag-NPs) may have several applications, such as catalysts in chemical reactions [3], electrical batteries and in spectrally selective coatings for absorption of solar energy [4,5], as optical elements [6], pharmaceutical components and in chemical sensing and biosensing [7,8]. Nanoparticle formation has been reported using chemical and physical methods. There are various methods for Ag-NPs formation such as sol-gel process, chemical precipitation, reverse micelle method, hydrothermal method, microwave, chemical vapour deposition and biological methods, *etc* [9,10,11]. 

Recently, biosynthetic methods have been investigated as a new way for the production of Ag-NPs. Biological methods are currently gaining importance because they are eco-friendly, cost effective, and don’t involve the use of any toxic chemicals for the synthesis of nanoparticles [12,13,14]. The biosynthesis of inorganic nanomaterials has been performed using eukaryotic organisms such as fungi to produce nanoparticles of gold and silver [15,16]. Synthesis of nanosilver particles using ascorbic acid and citrate as reducing agents has recently been reported [17]. An earlier study showed that *Shewanella* algae was found able to reduce gold ions, forming 10–20 nm gold nanoparticles [18]. 

There have been several reports on the synthesis of Ag-NPs using medicinal plants such as *Basella alba*, *Helianthus annus*, *Saccharum officinarum*, *Oryza sativa*, *Sorghum bicolour*, *Zea mays* [19], *Aloe vera* [20], *Medicago sativa* (Alfalfa) [21], *Capsicum annuum* [22], *Magnolia kobus* [23], *Cinnamomum camphora* leaf [24], and *Geranium* sp. [25] for pharmaceutical and biological applications. A green synthesis of nanosilver particles using a methanolic extract of *Eucalyptus hybrida* leaves was reported [26]. Another study related to the synthesis of nanoparticles from *Vitex negundo* L. leaf extract in water solution with heat treatment [27]. A profile of bioactive compounds of *Vitex negundo* has been reported, which revealed that the plant contained a high amount of total phenolic compounds and flavonoids, which are considered to be potent natural antioxidants [28,29]. 

Recently, some studies have shown that specially formulated Ag-NPs have good antibacterial activity [30]. The bacteria usually are incapable of developing resistance against Ag-NPs, because these nanomaterials can at the same time attack a broad range of targets in microorganisms such as proteins with thiol groups, cell walls and cell membranes. A recent study on *Escherichia coli* has shown that Ag-NPs react with cell walls and cytoplasmic membranes, resulting in pits in the cell wall of bacteria, and finally killing them [31]. It has been reported that greenly synthesized titania (TiO_2_) and silver nanocomposites (TANCs) can easily damage the cell walls of *E. coli* [32]. The antimicrobial and antiviral activity of silver ion, silver compounds and Ag-NPs have been thoroughly investigated in previous studies [33,34,35].

In this study, the synthesis and characterization of silver nanoparticles/*Vitex negundo* (Ag/*Vitex negundo*) by a green method reported. Ag-NPs were prepared using silver nitrate as silver precursor and methanol extract of *Vitex negundo* leaf as reducing agent and stabilizer. The antibacterial effect of Ag/*Vitex negundo* was evaluated against two pathogenic bacteria, including *Escherichia coli* (Gram negative) and *Staphylococcus aureus* (Gram positive) using the agar disc diffusion method. 

## 2. Results and Discussion

**Figure 1:**
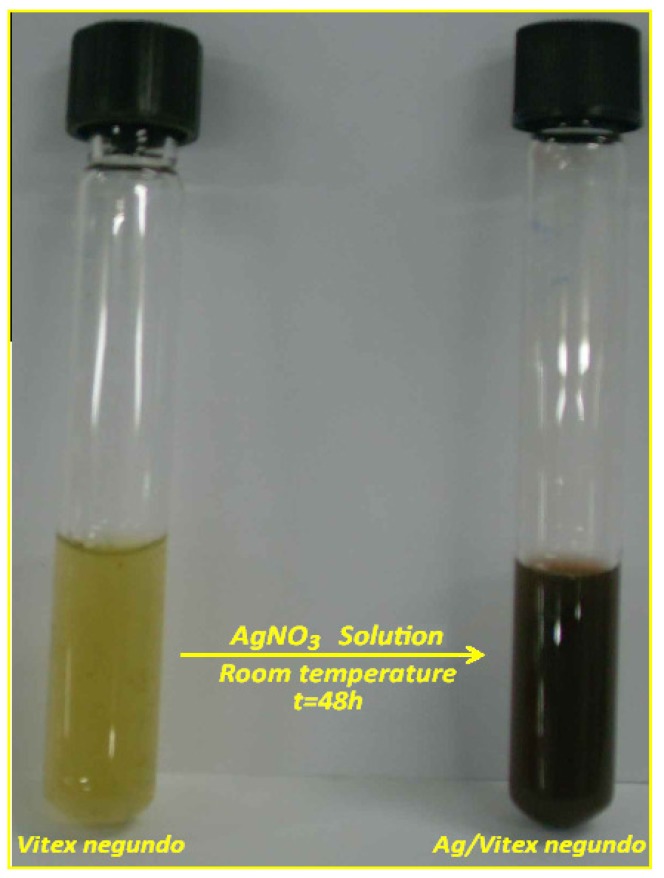
Photograph of (**a**) *Vitex negundo* and (**b**) Ag/*Vitex negundo* emulsion after 48 h.

Green synthesis of silver nanoparticles using 10^−1^ M, AgNO_3_ is shown in Figure 1. The fresh suspension of *Vitex negundo* was yellowish-green in colour. However, after addition of AgNO_3_ and stirring for 48 h at room temperature, the emulsion turned dark brown.

**Figure 2:**
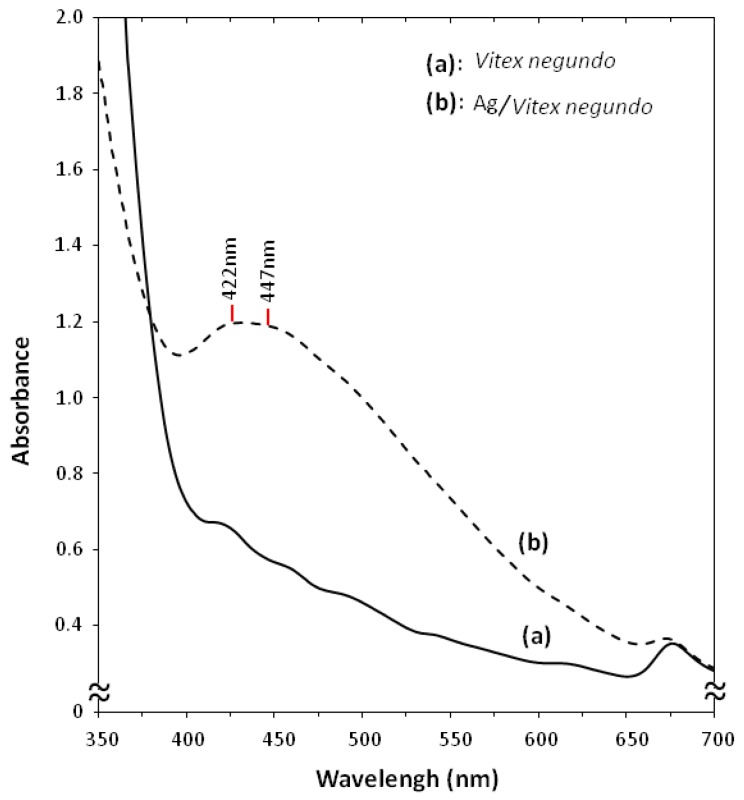
UV-Vis absorption spectra of (**a**) *Vitex* negundo and (**b**) Ag/*Vitex negundo* emulsion after 48 h.

The formation of silver nanoparticles was followed by measuring the surface plasmon resonance (SPR) of the *Vitex negundo* and Ag/*Vitex negundo* emulsion at the wavelength range from 300–700 nm (Figure 2). The characteristic silver SPR bands were detected around 400–450 nm. These absorption bands were assumed to correspond to the silver nanoparticles extra fine and smaller than 25 nm. UV–Vis absorption spectra (Figure 2b) showed that the broad SPR band contained two peaks, one at 422 nm and the other at 447 nm. These two peaks illustrate the presence of two broad distribution of hydrosol silver nanoparticles. 

**Figure 3:**
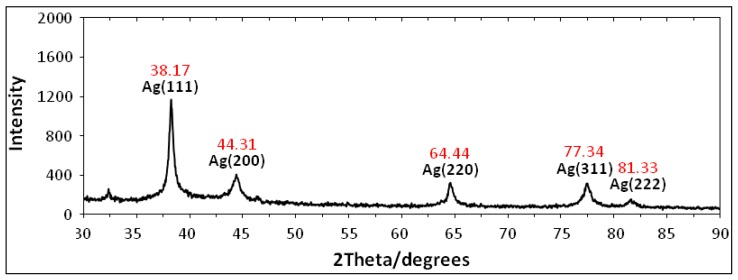
XRD patterns of Ag-NPs synthesized in *Vitex negundo* for determination of silver crystals after 48 h.

Figure 3 shows the X-ray diffraction (XRD) patterns of vacuum-dried silver nanoparticles synthesized using *Vitex negundo*. The XRD patterns of Ag/*Vitex negundo* indicated that the structure of silver nanoparticles is face-centered cubic (fcc) [36]. In addition, the silver nanoparticles had a similar diffraction profile and the XRD peaks at 2θ of 38.17°, 44.31°, 64.44°, 77.34° and 81.33° could be attributed to the 111, 200, 220, 311 and 222 crystallographic planes of the face-centered cubic (fcc) silver crystals, respectively [37].

The XRD pattern thus clearly illustrated that the silver nanoparticles formed in the study are crystalline in nature. The main crystalline phase was silver and there was no obvious other phases as impurities were found in the XRD patterns (Ag XRD Ref. No. 01-087-0719).

**Figure 4:**
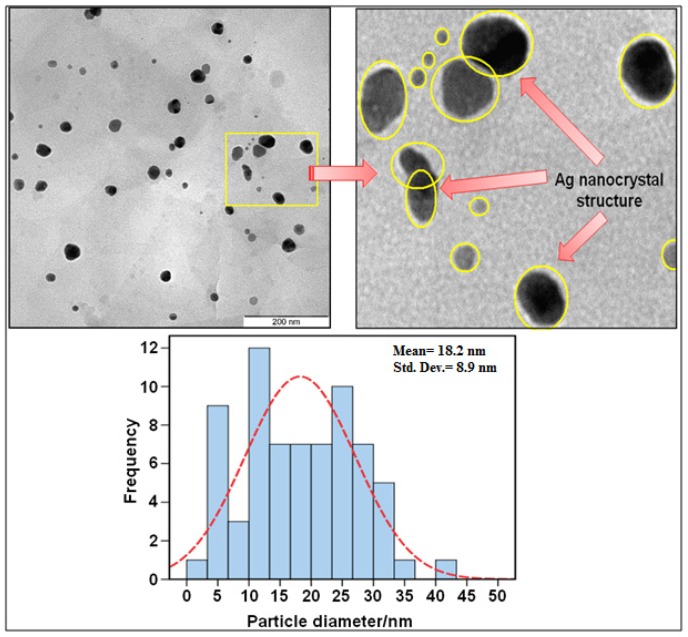
TEM image and corresponding size distribution of Ag/*Vitex negundo* after 48 h.

For the transmission electron microscopy (TEM) study, a drop of the silver nanoparticles solution synthesized by treating silver nitrate solution with *Vitex negundo* was deposited onto a TEM grid which was coated with carbon support film. After drying, this grid was imaged using TEM. Figure 4 shows a representative TEM image, with an enlargement on its right side. The TEM image and their size distribution showed that the main diameter and standard deviation of silver nanoparticles (18.2 ± 8.9 nm). The result showed two broad size distribution of particles, with diameter in the range of 10–20 nm and some larger diameter and uneven shapes in rang of 25–30 nm. The presence of two broad distributions of particles in TEM image is in accordance with the UV–Vis spectral study [38].

**Figure 5:**
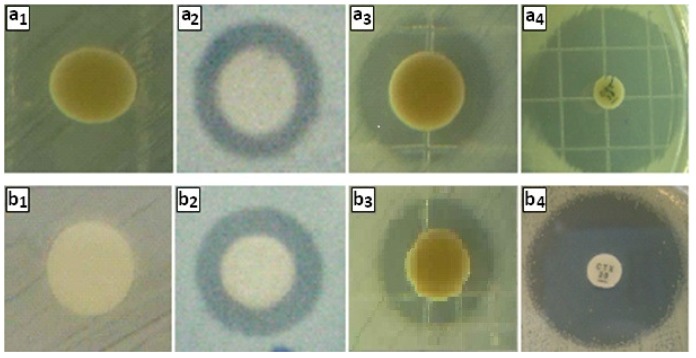
Antibacterial activity assay against *E. coli* (a_1_–a_4_) and *S. aureus* (b_1_–b_4_): (a_1_, b_1_) *Vitex negundo* extract (0.5 mg/mL), (a_2,_ b_2_) AgNO_3_ (0.17 mol/L), (a_3_, b_3_) Ag/*Vitex negundo*, (a_4_, b_4_) Cefotaxime (30 µg).

Growth inhibition zones values were obtained in millimeter for the synthesized Ag/*Vitex negundo* nanoparticles against *E. coli* and *S. aureus*. The results of the study presented in Figure 5 and Table 1. *Vitex negundo* extract (0.5 mg/mL) did not have any antibacterial effect (Figure 5a_1_ and 5b_1_). However, after formation of nanosilver, Ag/*Vitex negundo* emulsion has shown high antibacterial effect. The results presented in Figure 5a_3_ and 5b_3_ showed the antibacterial effects of Ag/*Vitex negundo* against *E. coli* and *S. aureus*, respectively. 

## 3. Experimental

### 3.1. Materials

Mature leaves of *Vitex negundo* were collected from the herbal unit of the Agriculture Park of Universiti Putra Malaysia (UPM). Methanol (CH_3_OH, 99.9%), AgNO_3_ (99.98%), nutrient agar and Mueller-Hinton agar (MHA) were purchased from Merck (Germany). All aqueous solutions were prepared using double distilled water. All reagents used were of analytical grade. 

### 3.2. Extract Preparation

*Vitex negundo* green leaves were washed and dried in an oven dryer at 40 °C for 48 h. The dried leaves were then ground into powder, stored in dark glass bottles and kept at −20 °C until further analyses. The finely ground *Vitex negundo* leaves (20 g) were extracted with methanol (ratio 1:10 w/v) overnight at 40 °C using a shaking water bath (Protech, Malaysia). After filtration with Whatman filter paper No 1 using vacuum pump, the residue was re-extracted. The solvent was completely removed using a rotary vacuum evaporator (Buchi, Flavil, Switzerland) at 40 °C. The concentrated extract was then kept in dark bottles at 4 °C until used. 

### 3.3. Synthesis of Ag/Vitex Negundo Emulsion 

Briefly, crude extract of *Vitex negundo* (0.5 g) was added to distilled de-ionized water (100 mL) with vigorous stirring for 1 h. A hundred milliliters of AgNO_3_ (1 × 10^−1^ M) was then added and mixed at room temperature (25 °C) for 48 h. Silver nanoparticles were gradually obtained during the incubation period. 

### 3.4. Evaluation of Antibacterial Activity

*In vitro* antibacterial activity of the prepared nanoparticles was evaluated using the Kirby-Bauer [39] technique, which conformed to the recommended standards of the National Committee for Clinical Laboratory Standards (NCCLS) (now known as Clinical and Laboratory Standards Institute CLSI). One species each of a Gram positive (*Staphylococcus aureus* ATCC 25923) and Gram negative bacteria (*Escherichia coli* ATCC 25922) were used for the antibacterial assay. Briefly, sterile paper discs (6 mm) impregnated with Ag/*Vitex negundo* (20 µL) were left to dry at 30 °C for 24 h in an incubator. Several isolated colonies of bacteria were selected from a culture of 12–18 h on nutrient agar (Merck, Germany) and dissolved in sterile saline. The suspension was adjusted to match the tube 0.5 McFarland turbidity standard using spectrophotometer in 600 nm, which equal to 1.5 × 10^8^ colony forming unit (CFU) per mL. The surface of MHA was completely cultured using a cotton swab which steeped in prepared suspension of bacterium. Finally, dried impregnated discs were placed on inoculated medium and incubated in 37 °C for 18–24 h. The diameter of zone inhibition was measured in millimeter, and was recorded as mean ± SD of the triplicate experiment. Cefotaxime (30 µg) was used as positive standard for comparison purposes.

### 3.5. Characterization Methods and Instruments 

The synthesized silver nanoparticles in *Vitex negundo* were characterized using X-ray diffraction, transmission electron microscopy and Ultraviolet-Visible spectroscopy. The structure of the silver nanoparticles was studied using the X-ray diffraction (XRD, Philips, X’pert, Cu Kα) at a scanning speed of 4°/min. The TEM observations were carried out using Hitachi H-7100 electron microscope, and the particle size distributions were determined using the UTHSCSA Image Tool version 3.00 programmed. The UV–Vis spectra were recorded over the range of 300–700 nm with a UV-visible spectrophotometer (H.UV 1650 PC-Shimadzu B.).

## 4. Conclusions

Nano-silver particles with an average size of 18.2 ± 8.9 nm and spherical shapes were synthesized using methanolic extract of *Vitex negundo* leaf. The Ag-NPs were characterized by UV-Visible, TEM and XRD measurements. Synthesis of Ag/NPs using green resources like *Vitex negundo* is a better alternative to chemical synthesis, since this green synthesis is pollutant free and eco-friendly. The results suggested that *Vitex negundo* plays an important role in the reduction and stabilization of silver to silver nanoparticles. Study also found that the Ag/*vitex negundo* shows antibacterial activity on both Gram positive and Gram negative bacteria and should be explored further for antimicrobial applications.

## Figures and Tables

**Table 1:** Average of inhibition zones synthesized Ag/*Vitex negundo* nanoparticles.

	Inhibition zone diameter (mm)			Control positive (mm)
Bacteria	*Vitex negundo* (0.5 mg/mL)	AgNO_3_ (0.17 mol/lit)	Ag/*Vitex negundo*	Cefotaxime (30 µg/disc)
*E. coli*	0.0	9.0 ± 0.5	12.0 ± 0.7	28.0 ± 0.5
*S. aureus*	0.0	9.5 ± 0.5	11.0 ± 0.3	21.0 ± 1.5
